# A Four-Step Purification Process for Gag VLPs: From Culture Supernatant to High-Purity Lyophilized Particles

**DOI:** 10.3390/vaccines9101154

**Published:** 2021-10-09

**Authors:** Irene González-Domínguez, Elianet Lorenzo, Alice Bernier, Laura Cervera, Francesc Gòdia, Amine Kamen

**Affiliations:** 1 Departament d’Enginyeria Química Biològica i Ambiental, Universitat Autònoma de Barcelona, 08193 Barcelona, Spain; Elianet.Lorenzo@uab.cat (E.L.); Laura.Cervera@uab.cat (L.C.); Francesc.Godia@uab.cat (F.G.); 2 Department of Bioengineering, McGill University, Montreal, QC H3A 0E9, Canada; bernier.alice@videotron.ca (A.B.); amine.kamen@mcgill.ca (A.K.)

**Keywords:** Gag VLPs, purification, clarification, chromatography, lyophilization, EVs, nanoparticle quantification

## Abstract

Gag-based virus-like particles (VLPs) have high potential as scaffolds for the development of chimeric vaccines and delivery strategies. The production of purified preparations that can be preserved independently from cold chains is highly desirable to facilitate distribution and access worldwide. In this work, a nimble purification has been developed, facilitating the production of Gag VLPs. Suspension-adapted HEK 293 cells cultured in chemically defined cell culture media were used to produce the VLPs. A four-step downstream process (DSP) consisting of membrane filtration, ion-exchange chromatography, polishing, and lyophilization was developed. The purification of VLPs from other contaminants such as host cell proteins (HCP), double-stranded DNA, or extracellular vesicles (EVs) was confirmed after their DSP. A concentration of 2.2 ± 0.8 × 10^9^ VLPs/mL in the lyophilized samples was obtained after its storage at room temperature for two months. Morphology and structural integrity of purified VLPs was assessed by cryo-TEM and NTA. Likewise, the purification methodologies proposed here could be easily scaled up and applied to purify similar enveloped viruses and vesicles.

## 1. Introduction

Virus-like particles (VLPs) are recognized as a promising strategy in recombinant vaccine development due to their ability to mimic native viruses with the lack of a viral genome. Their highly organized and repetitive antigen-presenting structure has been shown to stimulate both cellular and humoral immune responses [1,2]. Moreover, their easy and flexible production, based on recombinant protein expression, makes them an excellent nanocarrier for the delivery of immunogens, proteins, enzymes, and DNA in vitro and in vivo [1,3]. Among the different candidates, Gag VLPs offer great promise in the development of HIV-1 vaccine candidates [4] and as a scaffold for chimeric VLPs against other diseases [1]. The production of Gag VLPs needs the expression of the Gag polyprotein from the HIV-1 in a producer cell line. Upon expression, Gag travels to the cell membrane of the producer cell and after an oligomerization process, VLPs bud to the cell culture supernatant. Thus, the final nanoparticles are enveloped by a host cell lipid membrane [5], with an expected size between 100 and 200 nm.

Although the generation of these particles has been described in the literature for more than three decades, there are still challenges for their clinical-grade production [4,6]. Gag VLPs are normally produced in animal cell cultures, and thereafter, a bench-scale purification is performed. Centrifugation followed by ultracentrifugation is typically used to separate the cell culture supernatant from cells and to further concentrate the VLPs [4]. However, these methods are non-scalable and labor-intensive, which may cause inter-experimental variabilities. Furthermore, due to their enveloped structure, Gag VLPs are more sensitive to shear stress, osmotic pressure, and extreme pH than non-enveloped nanoparticles [4,6]. To overcome these problems, efforts are being undertaken toward the development of novel purification processes [4,7,8]. New matrices have been developed to maximize purification yields of viral structures by reducing the mechanical stress, increasing the pore size, and using more inert materials [6,8]: polymer-grafted beads, monoliths, membrane adsorbers, gigapore, heparin affinity, or mixed-mode resins in chromatographic steps are some examples [7,8,9,10,11,12,13].

Another factor that contributes to this challenging purification is the need for appropriate analytical methods to corroborate the structural integrity of the VLPs, as well as its separation from other nanoparticles [14]. Alongside with the classical contaminants encountered in cell culture-derived products, Gag VLPs share similar physicochemical and biochemical properties with the naturally secreted extracellular vesicles (EVs), limiting its discrimination within the same sample. Of note, the separation of these two subpopulations has not been described, although the enrichment of VLPs over EVs has been reported by some authors [13,15,16]. In previous works, we developed a fluorescent VLP by fusing the enhanced green fluorescence protein (eGFP) to the Gag polyprotein [17]. By doing so, nanoparticle tracking analysis (NTA) and flow virometry methods coupled to fluorescence have proven the simultaneous quantification of VLPs and EVs [18]. Compared to other biological and biochemical methods, these biophysical assays are not only able to distinguish between VLPs and EVs but also to specifically quantify assembled VLP structures from free Gag monomers released during cell culture death [4,18,19].

Finally, the preservation of purified Gag VLPs is another general concern in the field. While cold chain storage is still the classical approach, the instability associated with this step prior to administration can affect the efficiency of the candidate [20]. Engineering VLPs at the protein level [1] or the optimization of formulation buffer by the addition of sugars [21] are some reported solutions. Nonetheless, less information is available about the thermostability of Gag VLPs, and more appealing strategies such as freeze-drying have not been tested [22].

In this work, the stability, purification, and preservation of Gag VLPs have been studied. The combination of new membrane-based filtration and chromatographic methods together with lyophilization is proposed here. Specifically, a purification process consisting of depth filtration to obtain the clarified supernatant, ion-exchange chromatography (IEX) chromatography to concentrate the VLPs, and size-exclusion chromatography (SEC) to polish and prepare the purified material for lyophilization has been developed. VLP, EV, double-stranded DNA (dsDNA), and total protein concentration were quantified during the different unit operations. The obtained data on Gag VLP purification and preservation will contribute to define strategies that will facilitate their manufacturing and distribution worldwide [22].

## 2. Materials and Methods

### 2.1. Mammalian Cell Line, Culture Conditions, and Transient Gene Expression

The mammalian cell line used in this work is a serum-free suspension-adapted HEK 293 cell line (HEK 293SF-3F6, NRC, Montreal, QC, Canada). Cells were cultured in disposable PETG flask (Thermo Fisher, San Jose, CA, USA) at 37 °C, 5% CO_2_, 80% relative humidity at 110 rpm in Multitron shakers (INFORS HT, New York, NY, USA) with HyCell™ TransFx-H medium from HyClone™ (GE HealthCare, Chicago, IL, USA). HyCell™ culture medium was supplemented with 0.1% Kolliphor188 (Sigma-Aldrich, St Louis, MO, USA) and 4 mM GlutaMax (Gibco, Life Technologies, Thermo Fisher). Cell density and viability were routinely assessed with Vi-Cell XR (Beckman Coulter Inc., Brea, CA, USA).

HEK 293 cells were transfected using 25 kDa linear polyethylenimine (PEI) (PolySciences, Warrington, PA, USA) as previously described [23]. Gag-eGFP VLPs were obtained using transient gene expression with a pGag-eGFP plasmid, which codes for a Rev-independent Gag protein fused in frame to the enhanced GFP [15]. Shortly, the (HBX2) Gag DNA with the Kozak consensus sequence was synthesized by TOPGene Technologies (Sant-Laurent, QC, Canada) and cloned into a pAdCMV5-GFPq to give rise to the plasmid pAdCMV5-gagGFP encoding for the Gag-eGFP polyprotein. Gag-eGFP VLPs were harvested at 72 h post-transfection (hpt), ensuring cell culture viabilities higher than 90% [19]. Non-transfected negative controls reproducing cell growth conditions were also analyzed for comparison.

### 2.2. Spectrofluorometry

Quantification of Gag-eGFP in sample supernatants was assessed using an in-house developed and validated spectrofluorometry assay [17]. Green fluorescence was measured at room temperature (RT) using a Cary Eclipse Fluorescence Spectrophotometer (Agilent Technologies, Santa Clara, CA, USA) with the following settings: λex = 488 nm (slit 5 nm), λem = 510 nm (slit 10 nm). Relative fluorescence units (RFU) were calculated by subtracting fluorescence units (FU) values from non-transfected samples. There is a linear correlation between fluorescence intensity and p24 values determined using the HIV INNOTEST ELISA (Innogenetics NV, Gent, Belgium). RFU values can be converted to Gag-eGFP concentration values using the following Equation (1):

Gag-eGFP (ng/mL) = (3.245·RFU − 1.6833) × 36(1)

where Gag-eGFP is the estimated concentration of polyprotein and RFU is the measured GFP fluorescence intensity in the samples. RFU values from different days were normalized with a 0.1 mg/mL quinine sulfate solution as an internal control. To further convert Gag-eGFP concentration to VLP concentration, it was assumed that one VLP contains 2500 Gag-eGFP monomers with a molecular weight of 84 kDa per monomer [24].

### 2.3. Flow Virometry

Flow virometry experiments were performed with a CytoFLEX (Beckman Coulter Inc.) with violet side scatter (V-SSC) 405 nm filter configuration. In all cases, samples were diluted with PBS 1 X until the abort rate value was below 2%. A total of 300,000 events per sample were analyzed at a flow rate of 10 µL/min. V-SSC vs. B525-FITC density plots were used to gate the different particle populations. Gating was manually adjusted for each channel. The results were analyzed with the CytExpert software (Beckman Coulter). Nanoparticle concentrations were calculated with Equation (2):

(2) $$\frac{VLPs\;or\;EVs}{mL}=\frac{events}{uL}\cdot \frac{uL}{mL}\cdot Dilution$$

### 2.4. Total Protein, Host Cell Protein (HCP), and Double-Stranded DNA (dsDNA) Quantification

Total protein concentration was determined using RC/DC assay (Bio-Rad Laboratories, Hercules, CA, USA) or Micro BCA assay (Thermo Fisher) following the manufacturer’s instructions. The calibration curve was obtained using bovine serum albumin (BSA) standards (Thermo Fisher) diluted in distilled water with a concentration range of 50–250 µg/mL. The concentration of HEK293 HCP was determined by the HEK293 host cell protein ELISA kit (Cygnus, Southport, NC, USA) according to the manufacturer’s instructions. DsDNA quantification was performed with the Quant-iTTM PicoGreen^®^ dsDNA kit (Life Technologies). Since Gag-eGFP VLPs emit at the same range as the Quant-iTTM PicoGreen^®^ reagent, the native fluorescence was measured prior to the reagent addition and later subtracted to the fluorescence after the reaction. Assays were performed in a 96-well plate format. The 96-well plates were read and analyzed with Synergy HTX multi-mode reader and Gen5 software, respectively (BioTek Instruments, Inc., Winooski, VT, USA).

### 2.5. Dynamic Light Scattering (DLS)

DLS experiments were performed using a Zetasizer Nano ZS instrument (Malvern Instruments, Malvern, UK) with a He/Ne 633 nm laser at 173°. The hydrodynamic diameter and polydispersity index (PDI) were calculated with cumulative fit correlation at 25 °C and 0.8872 cP as previously described [25].

### 2.6. Nanoparticle Tracking Analysis (NTA)

Gag-eGFP VLPs and total particle content were analyzed by NTA. A NanoSight^®^ NS300 device was used (Malvern Panalytical, Malvern, U.K.) as previously described [18]. NanoSight^®^ NS300 is equipped with a blue laser module (488 nm) and a neutral density filter that was used to quantify GFP fluorescent nanoparticles and total particles by light diffraction, respectively. Gag-eGFP VLP concentrations were calculated as the total fluorescent particles, and the concentration of EVs was calculated as the difference between light scattering particles and fluorescent particles.

### 2.7. Cryogenic Transmission Electron Microscopy (Cryo-TEM)

Cryo-TEM analyses were performed at Servei de Microscòpia at Universitat Autònoma de Barcelona (UAB, Barcelona, Spain) using a Leica EM GP cryo workstation and observed in a Jeol JEM-2011 TEM electron microscope operating at 200 kV (JEOL USA, Pleasanton, CA, USA) as previously described [18].

### 2.8. Sodium Dodecyl Sulfate-Polyacrylamide Gel (SDS-PAGE) Electrophoresis and Western Blot Analysis

Precast TGX 4–15% Midi Gel (Bio-Rad Laboratories, Hercules, CA, USA) were used in a Tris-Glycine buffer system. The protocol was adapted from previous studies [26,27]. Briefly, samples were mixed with 1 × 1 Laemili buffer and 1% *v*/*v* of 1.4 M DTT. Each sample was incubated at 95 °C for 20 min. Precision Plus Protein™ Standards All Blue (Bio-Rad) was used as a protein marker. Samples were diluted 1:4 with distilled water before its addition to the gel. The gel was run at 200 V (400 mA). Coomassie staining solution was used to stain the total protein in the SDS-PAGE. For the western blot analysis, proteins were blotted using Trans-Blot^®^ turbo system (Bio-Rad) with 0.2 μm nitrocellulose membranes and blocked with 5% (*w*:*v*) skimmed milk overnight at 4 °C. Detection of Gag-eGFP protein was performed by incubation with primary mouse monoclonal antibody against HIV-1 p24 (ab9071, Abcam, Cambridge, UK), diluted 1:1000 in PBS-T for 2 h. Anti-mouse IgG conjugated with horseradish peroxidase (HRP) (Abcam), diluted 1:1000 in PBS-T was used as a secondary antibody. Finally, the membrane was incubated with enhanced chemiluminescence (ECL) reagent (Bio-Rad) and visualized using a ChemiDoc imager (Bio-Rad).

### 2.9. Thermostability Studies of Gag-eGFP VLPs

Preliminary thermostability studies of Gag-eGFP VLPs at different temperatures were performed prior to DSP development. Gag-eGFP VLPs were produced in HEK 293 cells by PEI-mediated transient gene expression as previously described [28]. Thermostability studies were performed during 90 days at four different conditions: 37, 4, −20, and −80 °C.

### 2.10. Preliminary Serial Filtration Experiments

Serial filtration experiments were performed with SartoScale PP3 polypropylene filter membrane of 25 mm and 4.5 cm^2^ effective surface area (Sartorius AG, Göttingen, Germany). Filters with 5, 3, 1.2, 0.65, and 0.45 µm nominal pore size were used. A total volume of 40 mL of cell culture supernatant containing Gag-eGFP VLPs were sequentially filtered at 266 LMH (liter per meter hour), corresponding to a flow rate of 2 mL/min at RT in sterile conditions with the different filters. MasterFlex^®^ pump with MasterFlex^®^ 96410-13 silicon tubes (Cole-Parmer, Montreal, QC, Canada) and pressure gauge (WIKA Alexander Wiegand SE & Co. KG, Klingenberg, Germany) connected to the filter inlet were used in the filtration experiments. A total of 2 mL of sample was taken between serial filtration for analysis. The same experiment was run for negative control for comparison.

### 2.11. Preparative Clarification Experiments

Preparative primary clarification of Gag-eGFP VLPs from HEK 293 cell cultures was carried out at 72 hpt. A total of 1 L of cell culture was incubated for 1.5 h at RT where flocculation of cells was obtained, followed by dead-end filtration with MilliStak^®^+ D0HC µpod filter with 23 cm^2^ effective surface area (Merck, Kenilworth, NJ, USA), adapted from [29]. Flocculation of cells was monitored through optical density at 600 nm, and the filtration process was monitored by measuring the eGFP fluorescence in the clarified material with Synergy HTX multi-mode reader and Gen5 software, respectively (BioTek Instruments, Inc.). Load capacity (L·m^−2^) of filters in the screening experiments was evaluated until feed pressure reached a maximum of 14.5 psi (1 bar). Filtration was performed at an average LMH of 135.5 ± 34.3 in sterile conditions. Filters were flushed and conditioned with 50 mL of 50 mM HEPES, pH 7.4, 300 mM NaCl buffer. MasterFlex^®^ pump with MasterFlex^®^ 96410-13 silicon tubes (Cole-Parmer) and pressure gauge (WIKA Alexander Wiegand SE & Co. KG) connected to the filter inlet were used for the filtration experiments.

### 2.12. Ion-Exchange Chromatography (IEX)

Clarified cell culture supernatant containing Gag-eGFP VLPs was loaded into a 0.86 mL XT Acrodisc^®^ Mustang^®^ QXT membrane adsorber (Mustang Q, PALL Corporation, New York, NY, USA). Before loading, the column was equilibrated with 50 mM HEPES, 100 mM NaCl, pH 7.2 (5% buffer B). After the loading phase, the column was washed with equilibration buffer (5% buffer B) for 5 membrane volumes. In the linear-gradient purification, a salt linear gradient from 100 to 1000 mM NaCl (5% to 50% buffer B) in 20 membrane volumes was used adapted from [27]. For the step gradient chromatography experiments, a final optimized method of 300, 700, 900, and 1200 mM (15%, 35%, 45%, and 60% buffer B) of 10 membrane volumes each was used. In both purification strategies, the column was regenerated with 100% buffer B for 10 membrane volumes. After regeneration, the membrane was sanitized using 10 membrane volumes of 1 M NaOH. UV absorbance at 280, 260, and 488 nm and pH were measured online in AKTA Explorer (GE Healthcare). All the preparative purification runs were performed using a flow rate from 1 to 5 mL/min in the flowthrough and of 1 mL/min in the elution. Fractions were collected and pooled according to the chromatograms.

### 2.13. SEC Chromatography

Polishing step after IEX at bench scale was performed with pD10 desalting column according to manufacturer’s instructions (GE Healthcare). Gag-eGFP VLPs were eluted in PBS (Hyclone). Preparative chromatography was performed with a 43 mL sepharose 4 Fast Flow (4FF) packed column at a constant flow rate of 1 or 2 mL/min. Briefly, SEC column was equilibrated with 20 mM NaH2PO4, 50 mM NaCl, 2 mM MgCl_2_, 2% sucrose at pH 7.5. Then, 4–6.4 mL of Gag-eGFP VLPs were loaded and eluted with the equilibration buffer in the void volume. UV absorbance at 280, 260, and 488 nm and pH were measured online in AKTA Explorer (GE Healthcare). The different fractions obtained were pooled and analyzed according to the chromatogram.

### 2.14. Lyophilization

Purified Gag-eGFP VLPs were aliquoted in 2 mL glass vials (Canadian Life Science, Peterborough, ON, Canada) for their lyophilization. The purified Gag-eGFP VLPs were previously buffer exchanged into lyophilization buffer (20 mM NaH2PO4, 50 mM NaCl, 2 mM MgCl_2_, 2% sucrose at pH 7.5). Aliquots were frozen at −80 °C and subsequently lyophilized at −60 °C at 100 µbar for 24 h in a VirTis BenchTop 6K (SP Industries, Warminster, PA, USA). Lyophilized material was stored at RT for two months until analysis. Lyophilized samples were resuspended in distilled water prior to analysis. Cryo-TEM, DLS, flow virometry, and NTA were used to evaluate particle integrity in purified samples.

## 3. Results

### 3.1. Stability Studies on Gag VLPs

**Figure 1:**
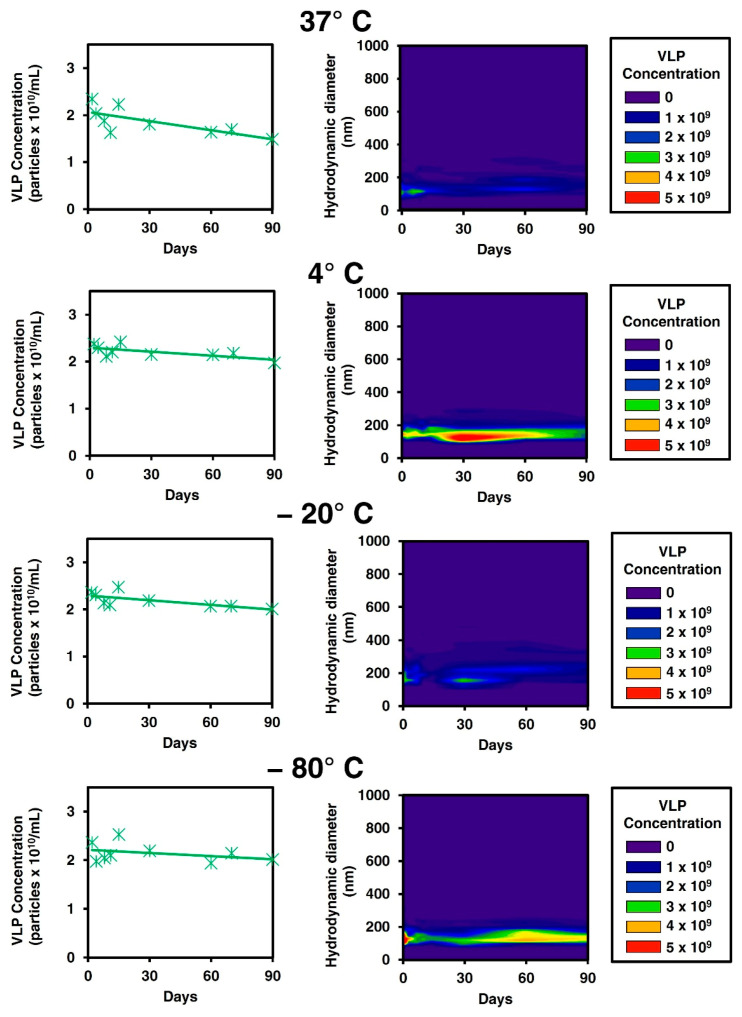
Gag-eGFP VLP thermostability studies. Gag-eGFP VLPs were stored at different temperatures: 37, 4, −20, and −80 °C, respectively. Concentration of Gag-eGFP VLPs was quantified by spectrofluorometry-based assay (line chart), and the particle size distribution (PSD) was analyzed with NTA (contour chart) for the four conditions with time.

The thermostability and aggregation of Gag VLPs were firstly studied to assess their resistance to different storage conditions and prevent further purification loss during the different unit operations. In the first experiment, cell culture temperature (37 °C), standard cold chain (4 °C), and freezing temperatures (−20 and −80 °C) were tested. Two different techniques were combined to analyze VLP stability over time: spectrofluorometry and fluorescent nanoparticle tracking analysis (NTA) (Figure 1). Spectrofluorometry enables the quantification of Gag-eGFP polyprotein that can be indirectly related to VLP concentrations [17], while NTA tracks individual GFP fluorescent nanoparticles [30]; thus, the specific quantification of assembled VLP from free Gag monomer and the particle size distribution (PSD) of the VLP population could be assessed, simultaneously.

An initial concentration of 2.4 ± 0.5 × 10^10^ VLPs/mL was quantified in crude harvested supernatants by both techniques (Figure 1). Along with storage, a constant VLP concentration of 2.4 ± 0.5 × 10^10^ and 2.2 ± 0.6 × 10^10^ VLPs/mL was observed at 4 or −80 °C by NTA over three months, respectively. Contrarily, a loss in VLP titer of 26% ± 11% and 52% ± 13% at 37 °C was measured by spectrofluorometry and NTA, after ten days, respectively. Thus, a degradation of the VLP structure and the Gag-eGFP polyprotein might be taking place in this condition. For −20 °C storage, a mean concentration of 2.2 ± 0.2 × 10^10^ VLPs/mL (0% loss) was obtained in Gag-eGFP quantification, while a loss of 47% ± 16% was quantified by NTA after 10 days of storage. The PSD analysis of −20 °C condition showed a loss of nanoparticles in the range of 100 to 200 nm and the appearance of larger aggregates with time (Figure 1). Since Gag-eGFP by fluorescence quantification is maintained, a particle disruption might be the main cause of instability at −20 °C due to a heterogeneous freezing process and the appearance of large ice crystals.

Sequential filtration experiments were performed in harvested Gag-eGFP VLPs to study whether these particles aggregate with other cell medium components upon production. A total of five membrane filters with 5, 3, 1.2, 0.65, and 0.45 µm pore size were tested. The same experiment was performed with a conditioned cell culture medium as well as from non-transfected supernatants. Analyses performed on VLP-containing supernatant (Figure S1A,B) or conditioned cell culture medium (Figure S1C) showed no significant loss of total protein, dsDNA, or VLP content. Furthermore, according to preliminary freezing experiments, up to two freeze and thaw cycles could be supported by Gag-eGFP VLPs at −80 °C since no concentration loss is observed compared to a non-frozen sample (data not shown). In summary, Gag-eGFP VLPs did not present any aggregation tendency in their native conditions and showed to be stable up to three months at 4 and −80 °C, whereas it is not recommended to store them at −20 °C degrees since a clear particle disruption is observed under this condition.

### 3.2. Development of Four Steps Downstream Process of Gag VLPs

A purification train of four-step consisting of filter-based clarification, IEX chromatography to concentrate the VLPs, and SEC to polish and prepare the purified material for lyophilization was developed, characterized, and validated.

#### 3.2.1. Clarification

**Figure 2:**
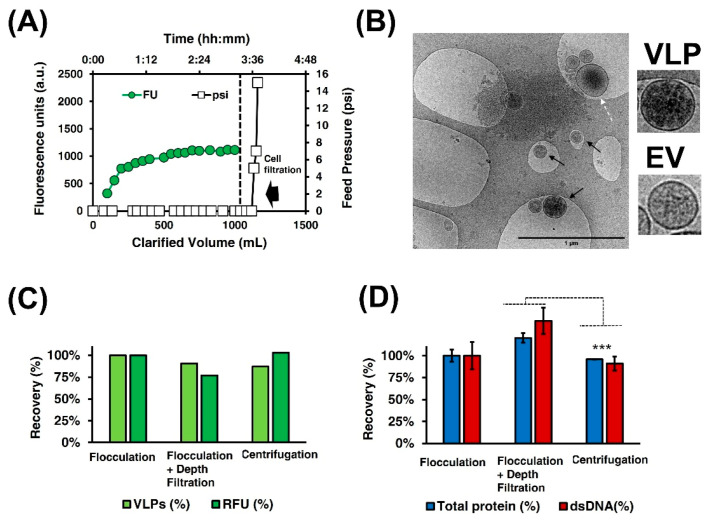
Clarification of Gag-eGFP VLPs. (**A**) At-line monitoring of depth filtration process, fluorescence units (FU) in the clarified volume, and feed pressure (psi) were measured with time. (**B**) Cryo-TEM micrographs of clarified material after filtration. Black arrows indicate the presence of VLPs, while white dashed arrows point to the EVs. Flocculation, flocculation and depth filtration, and centrifugation as the reference clarification strategy were analyzed (**C**,**D**); VLP measured by flow virometry (VLPs), relative fluorescent units (RFU) (**C**), total protein and dsDNA (**D**) recoveries (%) in respect to flocculation are compared between processes, respectively. Statistical analyses of technical replicates were performed with the T Student test in total protein and dsDNA quantifications. Ns: non-significant, ***: *p*-value ≤ 0.001.

Clarification aimed to separate cells from cell culture supernatant, where Gag-eGFP VLPs have been secreted during the production phase. To do so, a combined strategy encompassing flocculation and depth filtration was proposed. One liter of cell culture supernatant was incubated in a 1 L Erlenmeyer flask for 1.5 h at RT, where cells settled to the bottom of the flask, and the liquid phase was subsequently filtered. Due to the mean size diameter of these cells, which is around 15–20 µm [31], the Stokes law describes a very low gravity sedimentation velocity (*v*) calculated as follows:

(3) $$v=\frac{d^{2}\left(\rho_{p}-\rho_{m}\right)\times g}{18\;n}$$

where *g* is the acceleration of gravity (9.807 m/s^2^), $\rho_{p}$ is the particle density, $\rho_{m}$ is the medium density, *d* is the particle diameter, and n is the medium viscosity. Assuming a similar $\rho_{p},$ as those calculated for CHO cells, of 1051 kg/m^3^ [32], a $\rho_{m}$ of 1000 kg/m^3^,and a *n* of 0.001 kg/(m s) at 20 °C, a settling velocity in the order of few cm per hour is obtained from Equation (3) [33]. Thus, the cell broth sedimentation in a 1 L Erlenmeyer is expected to occur in several hours. Nonetheless, a settled bed was obtained after 1.5 h, pointing then spontaneous flocculation as the main driving force in the settling process. After that time, depth filtration with MilliStak^®^D0HC was performed. Feed pressure and fluorescence in the clarified volume were monitored during the experiment (Figure 2A). A total time of 3.5 h was needed to filtrate all the liquid phases, with no feed pressure observed during the process (~0 psi). Purification yield and contaminant removal were compared when only flocculation, flocculation and depth filtration, and centrifugation, as the reference protocol, were assessed (Figure 2C,D). A concentration of 8.2 × 10^9^, 7.4 × 10^9^, and 7.1 × 10^9^ VLPs/mL was calculated for the three processes, respectively (Figure 2C), while the dsDNA content and total protein were higher in the filtered sample compared to the centrifuged one (Figure 2D, *p*-value < 0.01). The clarified material was further characterized by cryo-TEM where the presence of VLPs (black arrows), but also the naturally co-produced EVs of different sizes were identified (white dashed arrows, Figure 2B).

As a proof of concept, settled cells were resuspended with the addition of 50 mL of PBS (Hyclone) and loaded to the filter. The addition of concentrated cells to the depth filter immediately increased the feed pressure to 16 psi (1.1 bar), over the defined limit of 1 bar. Thus, the settling of cells prior to depth filtration allowed to increase the filter capacity in an unlimited way since feed pressure only increased when cells were loaded. Similar improvements in terms of filter capacity were reported by Westoby and co-workers when CHO cells flocculation at acidic pH was performed before microfiltration [33].

#### 3.2.2. IEX Chromatography

**Figure 3:**
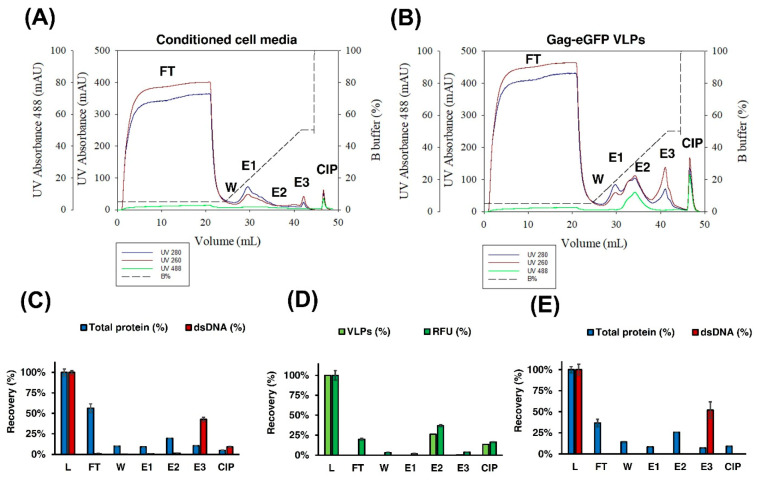
IEX chromatography purification of Gag-eGFP VLPs. (**A**,**B**) Chromatogram of the linear-gradient purification of conditioned medium or Gag-eGFP VLP supernatant using a Mustang^®^ QXT, respectively. The recovery of VLP nanoparticle concentration measured by flow virometry (VLPs) and relative fluorescent units (RFU) (**D**) and total protein and dsDNA content in the conditioned cell culture medium run (**C**) and in the Gag-eGFP VLP run (**E**) were also assessed. L: load material; FT: flowthrough; W: wash; E1-E3: pooled fractions for peaks 1–3 and CIP: cleaning in place with 2 M NaCl.

The concentration and purification of Gag-eGFP VLPs were assessed by means of IEX chromatography. Initial linear-gradient experiments were performed with clarified supernatant to evaluate the suitability of the Mustang Q membrane to purify these nanoparticles. A total of 20 mL of conditioned medium, as negative control and Gag-eGFP VLP-containing supernatant, were loaded into a 0.86 mL Mustang Q in two separate experiments (Figure 3A,C, respectively). Elution was assessed with a salt linear gradient from 100 to 1000 mM NaCl. Three different absorbances were followed online: UV260, UV280, and UV488 to monitor nucleic acid, protein, and Gag-eGFP content, respectively. The purification of Gag-eGFP VLPs from process-related impurities was clearly observed in these analyses, with a unique peak in UV488 absorbance (E2) in the VLP run compared to the negative control. This VLP peak could be separated from two other peaks detected in the UV260 and UV280 chromatograms, named E1 and E3, respectively. The VLP peak had an area of 496.1 mL·mAU measured with the UV488 absorbance that corresponds to 22.5–43.3% of B buffer and 26.7–61.5 mS/cm of conductivity (Figure 3C). The presence of Gag-eGFP VLPs was confirmed by flow virometry (VLPs) and RFU quantifications, where VLPs were recovered in the peak E2 (Figure 3D). The VLP peak presented a total protein concentration of 0.19 µg/mLm and dsDNA below the limit of detection (LOD).

From the total protein loaded, 37% ± 5% and 14% ± 0% were collected in the flowthrough (FT) and wash (W) fractions, respectively, while the rest was distributed in the other two peaks (E1 and E3). In the case of dsDNA, 52% ± 10% was recovered in the third peak (E3); meanwhile, it was below LOD in the other pooled fractions (Figure 3E). The same behavior was observed when only the conditioned medium was loaded (Figure 3B). Thus, the purification of Gag-eGFP VLPs is demonstrated by IEX NaCl salt gradient, where free proteins are collected in the FT and in the low-salt concentrations peak (E1), while the dsDNA is strongly bound to the membrane and is eluted at higher salt concentrations (E3).

**Figure 4:**
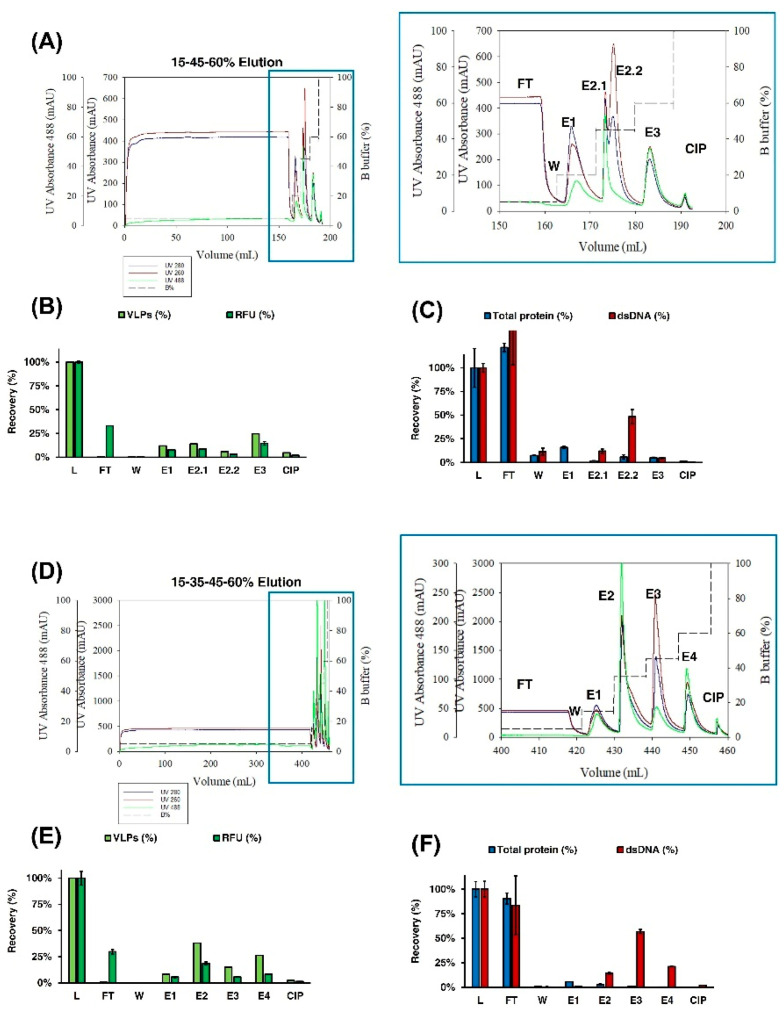
Optimization of IEX chromatography. (**A**,**D**) Chromatogram of the step gradient purification of Gag-eGFP VLP supernatant using a Mustang^®^ QXT. A total load of 150 mL was eluted in a step gradient of 15%–45%–60% of B buffer (**A**), or a total load of 413 mL was eluted in a step gradient of 15%–35%–45%–60% of B buffer (**D**), respectively. The recovery of VLP nanoparticle concentration measured by flow virometry (VLPs) and relative fluorescent units (RFU) (**B**,**E**) and total protein and dsDNA content (**C**,**F**) were also assessed, respectively. L: load material; FT: flowthrough; W: wash; E1-E3: pooled fractions for peaks 1–3 and CIP: cleaning in place with 2 M NaCl.

Optimization of the IEX chromatography step was performed, focusing on two main aspects: studying the dynamic binding capacity (DBC) and optimizing the elution by applying a step gradient to concentrate the Gag-eGFP VLPs in the elution phase. A total volume of 150 mL representing 7.5 times more supernatant than in the previous run was loaded to study the DBC. Thereafter, an initial three-step gradient of 15%, 45%, and 60% of buffer B was tested, based on the results observed in the linear gradient (Figure 3C(E1–E3), respectively). The results are presented in Figure 4A–C. The absence of Gag-eGFP VLPs was observed in the FT (Figure 4B) when a total amount of 6.6 × 10^11^ VLPs was loaded, according to flow virometry results. A second run was then performed, with a total volume of 413 mL of supernatant loaded (Figure 4E–G). In this case, the presence of Gag-eGFP VLPs was neither observed in the FT from a total load of 3.33 × 10^12^ VLPs. Thus, a load of 3.84 × 10^12^ VLPs/mL of Mustang Q membrane and 480 mL per mL of membrane volume was obtained in the absence of VLP breakthrough in the flowthrough.

Regarding the elution, two peaks were found in the 45% step corresponding to Gag-eGFP VLPs and a second corresponding to dsDNA (Figure 4A–C (E2.1 and E2.2), respectively). Therefore, an alteration of the VLP purification profile was observed when higher amounts of supernatant were loaded, likely because of the strong DNA binding with the QA membrane. To separate this double peak obtained in the 45% fraction, a four-step elution profile was then assessed encompassing 15%, 35%, 45%, and 60% of B buffer (Figure 4E–G). With this new profile, Gag-eGFP VLPs were concentrated in the 35% fraction with a concentration of 4.22 × 10^11^ VLPs/mL and a total recovery of 38% of the VLPs (Figure 4E). This recovery represents a VLP concentration of 56-fold and volume reduction of 55-fold compared to crude supernatants. Likewise, dsDNA was eluted in the 45% fraction, and almost all total protein was removed with the flowthrough, as expected from previous runs (Figure 4F).

Of note, the presence of Gag-eGFP nanoparticles was also observed in 15% and 60% salt steps representing 8% and 26% of the load material, respectively. To assess the presence of nanoparticles, E1, E2.1, and E4 peaks from the four-step run were loaded to a pd10 desalting column to remove the excess of NaCl in the elution fraction and were analyzed by DLS (S2A). In all three cases, a mean hydrodynamic diameter between 100 and 200 nm was found, such as in the loaded supernatant (S), whereas biochemical analyses confirmed Gag-eGFP presence in all samples (~87 kDa) by means of SDS-PAGE and Western blot anti-p24 (S2B and S2C, respectively).

#### 3.2.3. SEC Chromatography

**Figure 5:**
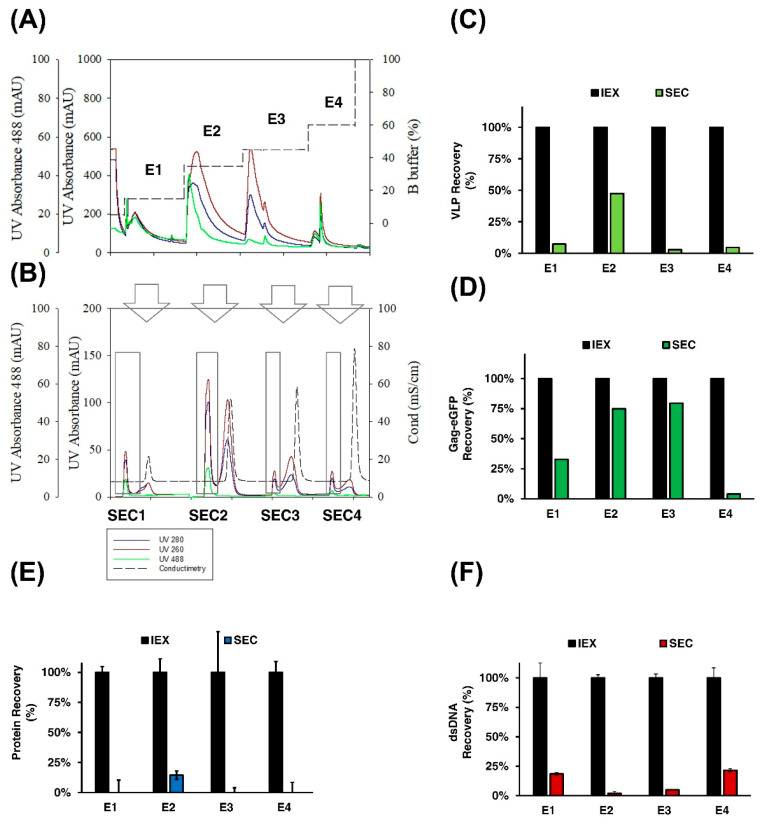
SEC of Gag-eGFP VLPs. (**A**) Chromatogram of IEX chromatography based on previous optimization protocol using a Mustang^®^ QXT. Gag-eGFP VLP supernatant produced in HEK 293 cell culture, clarified by flocculation and depth filtration was used as initial material. Equilibration buffer was 50 mM HEPES, 100 mM NaCl with pH 7.2, and step gradient of a total load of 283 mL eluted in step gradient of 15%–35%–45%–60% of B buffer corresponding to E1-E2-E3-E4 peaks. (**B**) Chromatogram of SEC from the four peaks obtained in IEX (SEC1-SEC2-SEC3-SEC4, respectively). A 43 mL Sepharose 4 Fast Flow (4FF) packed column was used. SEC column was equilibrated with 20 mM NaH2PO4, 50 mM NaCl, 2 mM MgCl_2_, 2% sucrose pH 7.5. The recovery of VLPs (**C**), Gag-eGFP polyprotein (**D**), total protein (**E**), and dsDNA (**F**) was assessed for each peak.

IEX fractions (Figure 5A, E1–E4) were loaded into a 43 mL SEC column, where VLPs were collected in the void volume diluted in lyophilization buffer (Figure 5B, SEC1–SEC4). The removal of NaCl coming from IEX was tracked with a conductimetry sensor (dotted line, Figure 5B). Average removal of 96% ± 7% and 88% ± 1% in terms of total protein and dsDNA was obtained after SEC in all the fractions (Figure 5E,F, respectively).

From the total Gag-eGFP VLPs loaded to SEC column, highly concentrated VLPs were only obtained from the 35% salt step of IEX (E2), as shown by the presence of a large peak of 1126 mL·mAU in SEC2 pooled fraction. This peak corresponded to 1.08 × 10^11^ VLPs and represented 75% of the Gag-eGFP polyprotein-loaded material (Figure 5D). As for the other IEX fractions, a VLP recovery amount of less than 10% was obtained, suggesting that marginal VLP amounts of correctly assembled particles are recovered in these fractions compared to the E2 fraction.

To validate these results, a complete purification process was performed, combining the clarification, IEX, and SEC steps. The mass balance of this complete validation run is summarized in Table 1 and Table 2. Overall, a total recovery of 23% of Gag-eGFP VLPs was achieved with a total protein, HCP, and dsDNA removal of almost 100% from the crude supernatant, presenting a final concentration of 38.5 µg/mL, 3.1 µg/mL, and 31.8 ng/mL of total protein, host cell protein, and dsDNA, respectively (Table 1). Of note, a VLP/total particles ratio of 56% was obtained after SEC, pointing to a notable enrichment of Gag-eGFP VLPs over EVs (Table 2).

#### 3.2.4. Lyophilization of Gag-eGFP VLPs

Finally, first attempts in the lyophilization of Gag-eGFP VLPs were performed in views to optimize its storage and transportation. Gag-eGFP VLPs were lyophilized in 1 mL aliquots stored at RT for two months. Alternatively, purified Gag-eGFP VLPs were frozen at −80 °C, in the same buffer, as the control.

**Figure 6:**
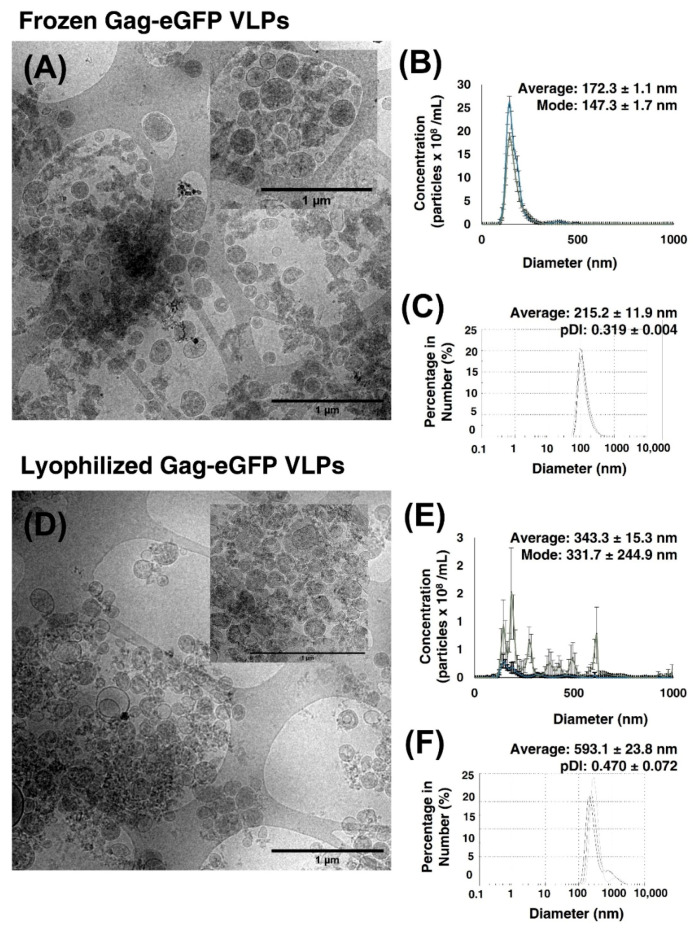
Biophysical characterization of purified Gag-eGFP VLPs. The purified nanoparticles were stored under different conditions for two months after SEC. (**A**–**C**) Frozen Gag-eGFP VLPs (**D**–**F**) Lyophilized Gage-GFP VLPs. (**A**,**D**) Cryo-TEM micrographs of the purified material, respectively. (**B**,**E**) PSD analysis by NTA of total nanoparticles (blue line) measured by light scattering or VLPs (gray line) measured with the fluorescence filter. Gag VLP concentration of 12.6 ± 0.7 × 10^9^ particles/mL and EV concentration of 9.9 ± 0.4 × 10^9^ particles/mL were obtained in frozen samples, whereas a VLP concentration of 2.2 ± 0.8 × 10^9^ particles/mL and an EV concentration below LOD was obtained after lyophilization (**C**,**F**) PSD analysis with DLS. Three independent measurements were performed. Average: mean hydrodynamic diameter; PDI: polydispersity index.

Concentrated and purified Gag-eGFP VLPs are clearly observed in cryo-TEM micrographs (Figure 6), compared to the initial clarified material depicted in Figure 2B. Gag-eGFP VLPs are detected as spherical electrodense nanoparticles surrounded by a lipid membrane in both frozen (Figure 6A) and lyophilized and reconstituted samples (Figure 6D). The particle size distribution (PSD) analyses showed a mean hydrodynamic diameter of 172.1 ± 1.1 nm and 215.2 ± 11.9 nm measured by fluorescent NTA or DLS in the frozen sample, respectively(Figure 6B,C), while nanoparticle aggregation was observed after lyophilization with a PSD of 343.3 ± 15.3 and 593.1 ± 23.8 nm measured with NTA and DLS, respectively (Figure 6E,F). Nanoparticle concentration of VLPs and EVs presented a decrease in VLP concentration after lyophilization, likely to the aggregation phenomenon since similar apparent concentrations are depicted in cryo-TEM analyses. Compared to crude clarified material (Figure 2E), the presence of EVs was very low in cryo-TEM micrographs and represented 2% of the initial material after SEC and was below LOD in lyophilized samples (Figure 6).

## 4. Discussion

### 4.1. Purification Train and Unit Capacity

A four-step process has been developed for Gag-eGFP VLPs. Load capacities and flux rates are summarized in Table 3. Load capacities between 400 and 800 L/m^2^ were achieved in membrane-based processes. Previous works analyzed different filters for primary and secondary clarification for influenza VLPs produced with baculovirus expression vector system (BEVS), where a maximum load of 150 L/m^2^ was reported for primary clarification with MilliStak^®^+ D0HC [29]. Here, the settling of cells before filtration allowed for the clarification of the whole-cell culture in one single run. Feed pressure was maintained at 0 psi, preventing possible VLP mechanical stress during clarification.

IEX chromatography was performed with a Mustang Q composed of 16 layers of Mustang Q membrane with a bed volume of 0.86 mL and a surface area of 4.9 cm^2^. From the different optimizations performed, a maximum load capacity of 842.9 L/m^2^ was achieved (Figure 4D), corresponding to 480.2 mL per mL of bed volume. Of note, DBC was not achieved in any of the experiments tested. Thus, a VLP load higher than 3.84·10^12^ VLPs is still possible, although maximum column pressure provided by the manufacturer or operation time might then be the limiting factor. High DBC of 4.70 × 10^10^ virus particles (vp) per square centimeter of adenoviruses, more than 8.7 × 10^10^ transduction units (TU) of lentiviral vectors, and 1.27 × 10^8^ infective units (ifu) of γ-retorviruses per mL of bed volume have been reported with membrane adsorbers [34,35,36]. Similarly, Steppert and co-workers reported a DBC of 8.6 × 10^12^ of total particles per mL in QA monolith in the purification of Gag VLPs [10]. These data support the use of strong quaternary amine ion exchangers to concentrate and purify enveloped viruses and viral particles, as those used in the present work. In addition, these novel materials work at faster flow rates by allowing convective flux inside the matrix [37]. On the other hand, due to the strong binding obtained with the Q ligand, high salt concentrations are required for the elution, and direct competition with dsDNA is observed. With views to overcome this fact, alternative weak ligands have also been proposed where dsDNA might be removed in the flowthrough [16].

SEC chromatography has been used as a desalting column after IEX chromatography to perform a buffer exchange. A maximum 6.4 mL load is allowed in a 43 mL SEC column for desalting purposes. Thus, several rounds are required to purify higher volumes, as for the E2 fraction here. Despite the simplicity of the method, a dilution factor of 1.7 and larger operation times are needed. Alternative membrane-based ultrafiltration and diafiltration have been described in the literature to overcome such limitations. Specifically, tangential flow filtration (TFF) with 300 and 1000 kDa cutoff membranes has been used for the concentration of influenza VLPs with higher recoveries [14,38]. Moreover, the use of hollow fibers has been highlighted in virus purification due to their open channels and lot-to-lot consistency [6,37]. Hence, the replacement of the SEC unit by another membrane-based method offers great promise to overcome the present constraints.

### 4.2. VLP Quality and Purification Yield

Lyophilized Gag-eGFP VLP vials containing 2.2 × 10^9^ VLPs have been purified from HEK 293 clarified supernatants. More than 30 µg/mL of Gag-eGFP polyprotein are obtained after purification, which represents the 98% of the total protein (Table 1). Of note, no nuclease treatment was added before IEX chromatography, and dsDNA was almost completely removed, resulting in a concentration of only 31.88 ng/mL before lyophilization. Furthermore, the quantity of EVs was reduced to 6% of the initial EV content (Table 2), whereas their quantification was below LOD by NTA after its lyophilization (Figure 6). The presence of EVs in the VLP preparation was almost neglectable in cryo-TEM micrographs. Although the enrichment of Gag VLPs over total particles has been reported previously [13,16,38], further analyses are required to confirm those results since indirect quantification methods have mostly been employed [4]. In this work, the presence of eGFP in fusion with the Gag polyprotein eases the specific quantification of assembled VLPs from total nanoparticles during the purification process.

Despite the obvious advantages of lyophilized material in comparison to cold chain storage, the process of freeze-drying is still a challenge in viral-based vaccines, especially for enveloped viruses. Lipid and protein damage, changes in the pH or osmolarity, or aggregation are some of the reported drawbacks [22]. Here, Gag-eGFP VLPs were lyophilized in a tailored lyophilization buffer, with preservation of particle integrity confirmed by cryo-TEM. Nonetheless, particle aggregation was observed with particle tracking analysis techniques. Together with the 2% (w:v) sucrose used in this work, the addition of sorbitol or gelatin as cryoprotectants might prevent membrane damage and aggregation. On the other hand, process parameters such as the freezing rate have also been considered critical to preventing ice formation during lyophilization [22]. Thus, further optimization of the formulation buffer and process parameters might improve these results.

Overall, there is no established process in the purification of viral-based products [7]. In a recent review, Moleirinho et al. showed different combinations of membrane-based and chromatographic purification with yields ranging from 20% to more than 90% recoveries [8]. Differences in composition and physicochemical properties demand tailored methods for each candidate. Cervera and coauthors reviewed different approaches for Gag VLPs [4]. The authors suggested a complete purification train for Gag VLPs consisting of depth filtration, benzonase treatment, IEX or TFF for concentration, and SEC or TFF for polishing followed by sterile filtration [4]. Here, a similar scalable DSP has been used in the absence of benzonase treatment, which is known to increase production costs.

A 23% recovery of highly purified VLPs from the crude supernatant was achieved (Table 2). While clarification showed no significant loss of Gag-eGFP VLPs, the elution of more than one peak containing Gag-eGFP VLPs was observed after IEX chromatography. The presence of different subpopulations within Gag VLPs has been described with the use of monolith and polymer-grafted matrices [11,16,27]. Similar recoveries from 20% to 50% have been reported in those works after IEX chromatography. Here, IEX chromatography allowed a selective purification of highly concentrated and homogenous Gag-eGFP VLPs [22], which might be desirable for clinical use. After SEC (Figure 5), the amount of VLPs recovered in P2 proved to be the highest, showing enrichment in correctly assembled particles (Figure 5). Alternative purification strategies based on TFF modules have been reported with recoveries of 44% and more than 80%*,* respectively [14,38]. However, higher levels of impurities with DNA concentration in the range of micrograms and higher total protein and HCP were observed compared to this work. Regarding the concentration factor achieved, 15 to 60 μg of antigen per dose have been described for VLP-based products [19]. Regulatory agencies established a limit of 10 ng of dsDNA and 100 ppm of HCP per dose in the final biological product [39,40]. Considering a dose of 10 μg of Gag-eGFP or 1 × 10^9^ VLPs, the present strategy meets the required quality attributes and offers great promise for the further use of this platform in the development of novel vaccine candidates.

## 5. Conclusions

Gag VLPs are a very promising platform for antigen and drug delivery strategies. A complete purification train from crude supernatants to highly purified Gag VLP preparations has been developed in this work. Thermostability and particle aggregation studies of harvested Gag-eGFP VLPs showed these particles to be stable at 4 and −80 °C for up to three months. A nimble DSP consisting of clarification, IEX chromatography, SEC, and lyophilization was developed. The separation of Gag-eGFP VLPs from EVs was observed in the final purified samples, where Gag-eGFP polyprotein represented more than 98% of total protein. In summary, lyophilized vials were obtained and stored at RT for up to two months. These preliminary results in enveloped VLP lyophilization open the window for developing new formulations increasing nanoparticle stability for its use and transportation worldwide.

## Figures and Tables

**Table 1:** Mass balance of total protein, HCP, dsDNA, and Gag-eGFP polyprotein. Cl: clarified material; IEX: E2 peak from EIX chromatography; SEC: SEC2 peak from SEC.

Samples	Volume (mL)	Total Protein		Host Cell Protein		dsDNA		Gag-eGFP Protein		
		Concentration (µg/mL)	Recovery (%)	Concentration (µg/mL)	Recovery (%)	Concentration (ng/mL)	Recovery (%)	Concentration (µg/mL)	Recovery (%)	Gag-eGFP/Total Protein (%)
CL	235	707.8	100	1.5	100	657.0	100	9.5	100	1
IEX	6	540.9	2	2.5	4	1610.7	6	99.1	27	18
SEC	10	38.5	0	3.1	9	31.8	0	37.8	17	98

**Table 2:** Nanoparticle mass balance of VLPs and EVs based on NTA quantification. Cl: clarified material; IEX: E2 peak from EIX chromatography; SEC: SEC2 peak from SEC.

Sample	Volume (mL)	NTA (Particles × 10^9^/mL)		VLP Recovery (%)	EV Recovery (%)	VLPs/ Total Particles (%)
		VLPs	EVs			
Cl	235	8.2	23.8	100	100	26
IEX	6	117.0	238.0	37	25	36
SEC	10	43.3	34.3	23	6	56

**Table 3:** Summary of process parameters.

Process	Maximum Volume (mL)	Effective Surface Area|Volume		Load Capacity		Concentration Factor	Flux Rate	
		Area (cm^2^)	Volume (mL)	(L/m^2^)	(mL/mL)		LMH	mL/min
Primary Filtration	1000	23	-	434.8	-	1	135.5	-
IEX Chromatography	413	4.9	0.86	842.9	480.2	50–55	-	1–5
SEC Chromatography	6.4	2.01	43	-	0.2	0.6	-	1–2

## Data Availability

Not applicable.

## Supplementary Materials

The following are available online at https://www.mdpi.com/article/10.3390/vaccines9101154/s1, Figure S1: Sequential filtration experiments of Gag-eGFP VLPs. Figure S2: Biophysical and biochemical characterization of purified Gag VLPs.

## References

[1] Charlton Hume H.K., Vidigal J., Carrondo M.J.T., Middelberg A.P.J., Roldão A., Lua L.H.L. (2019). Synthetic biology for bioengineering virus-like particle vaccines. Biotechnol. Bioeng..

[2] Donaldson B., Lateef Z., Walker G.F., Young S.L., Ward V.K. (2018). Virus-like particle vaccines: Immunology and formulation for clinical translation. Expert Rev. Vaccines.

[3] Hill B.D., Zak A., Khera E., Wen F. (2017). Engineering Virus-like Particles for Antigen and Drug Delivery. Curr. Protein Pept. Sci..

[4] Cervera L., Gòdia F., Tarrés-Freixas F., Aguilar-Gurrieri C., Carrillo J., Blanco J., Gutiérrez-Granados S. (2019). Production of HIV-1-based virus-like particles for vaccination: Achievements and limits. Appl. Microbiol. Biotechnol..

[5] Göttlinger H.G., Kuiken C.K., Foley B., Leitner T., Apetrei C., Hahn B., Mizrachi I., Mullins J., Rambaut A., StevenWolinsky S. (2001). HIV-1 Gag: A Molecular Machine Driving Viral Particle Assembly and Release.

[6] Besnard L., Fabre V., Fettig M., Gousseinov E., Kawakami Y., Laroudie N., Scanlan C., Pattnaik P. (2016). Clarification of vaccines: An overview of filter based technology trends and best practices. Biotechnol. Adv..

[7] Effio C.L., Hubbuch J. (2015). Next generation vaccines and vectors: Designing downstream processes for recombinant protein-based virus-like particles. Biotechnol. J..

[8] Moleirinho M.G., Silva R.J.S., Alves P.M., Carrondo M.J.T., Peixoto C. (2020). Current challenges in biotherapeutic particles manufacturing. Expert Opin. Biol. Ther..

[9] Pato T.P., Souza M.C.O., Silva A.N.M.R., Pereira R.C., Silva M.V., Caride E., Gaspar L.P., Freire M.S., Castilho L.R. (2014). Development of a membrane adsorber based capture step for the purification of yellow fever virus. Vaccine.

[10] Steppert P., Burgstaller D., Klausberger M., Berger E., Aguilar P.P., Schneider T.A., Kramberger P., Tover A., Nöbauer K., Razzazi-Fazeli E. (2016). Purification of HIV-1 gag virus-like particles and separation of other extracellular particles. J. Chromatogr. A.

[11] Pereira Aguilar P., Schneider T.A., Wetter V., Maresch D., Ling W.L., Tover A., Steppert P., Jungbauer A. (2019). Polymer-grafted chromatography media for the purification of enveloped virus-like particles, exemplified with HIV-1 gag VLP. Vaccine.

[12] Aguilar P.P., Reiter K., Wetter V., Steppert P., Maresch D., Ling W.L., Satzer P., Jungbauer A. (2020). Capture and purification of Human Immunodeficiency Virus-1 virus-like particles: Convective media vs. porous beads. J. Chromatogr. A.

[13] Reiter K., Aguilar P.P., Wetter V., Steppert P., Tover A., Jungbauer A. (2019). Separation of virus-like particles and extracellular vesicles by flow-through and heparin affinity chromatography. J. Chromatogr. A.

[14] Carvalho S.B., Silva R.J.S., Moleirinho M.G., Cunha B., Moreira A.S., Xenopoulos A., Alves P.M., Carrondo M.J.T., Peixoto C. (2019). Membrane-Based Approach for the Downstream Processing of Influenza Virus-Like Particles. Biotechnol. J..

[15] Venereo-Sanchez A., Gilbert R., Simoneau M., Caron A., Chahal P., Chen W., Ansorge S., Li X., Henry O., Kamen A. (2016). Hemagglutinin and neuraminidase containing virus-like particles produced in HEK-293 suspension culture: An effective influenza vaccine candidate. Vaccine.

[16] Steppert P., Burgstaller D., Klausberger M., Kramberger P., Tover A., Berger E., Nöbauer K., Razzazi-Fazeli E., Jungbauer A. (2017). Separation of HIV-1 gag virus-like particles from vesicular particles impurities by hydroxyl-functionalized monoliths. J. Sep. Sci..

[17] Gutiérrez-Granados S., Cervera L., Gòdia F., Carrillo J., Segura M.M., Gutierrez-Granados S., Cervera L., Godia F., Carrillo J., Segura M.M. (2013). Development and validation of a quantitation assay for fluorescently tagged HIV-1 virus-like particles. J. Virol. Methods.

[18] González-Domínguez I., Puente-Massaguer E., Cervera L., Gòdia F. (2020). Quality Assessment of Virus-Like Particles at Single Particle Level: A Comparative Study. Viruses.

[19] Cervera L., González-Domínguez I., Segura M.M., Gòdia F. (2017). Intracellular characterization of Gag VLP production by transient transfection of HEK 293 cells. Biotechnol. Bioeng..

[20] Kumru O.S., Joshi S.B., Smith D.E., Middaugh C.R., Prusik T., Volkin D.B. (2014). Vaccine instability in the cold chain: Mechanisms, analysis and formulation strategies. Biologicals.

[21] Lynch A., Meyers A.E., Williamson A.-L., Rybicki E.P. (2012). Stability studies of HIV-1 Pr55gag virus-like particles made in insect cells after storage in various formulation media. Virol. J..

[22] Hansen L.J.J., Daoussi R., Vervaet C., Remon J.P., De Beer T.R.M. (2015). Freeze-drying of live virus vaccines: A review. Vaccine.

[23] Cervera L., Gutiérrez-Granados S., Martínez M., Blanco J., Gòdia F., Segura M.M. (2013). Generation of HIV-1 Gag VLPs by transient transfection of HEK 293 suspension cell cultures using an optimized animal-derived component free medium. J. Biotechnol..

[24] Chen Y., Wu B., Musier-Forsyth K., Mansky L.M., Mueller J.D. (2009). Fluorescence fluctuation spectroscopy on viral-like particles reveals variable Gag stoichiometry. Biophys. J..

[25] González-Domínguez I., Grimaldi N., Cervera L., Ventosa N., Gòdia F. (2019). Impact of physicochemical properties of DNA/PEI complexes on transient transfection of mammalian cells. New Biotechnol..

[26] Joshi P.R.H., Cervera L., Ahmed I., Kondratov O., Zolotukhin S., Schrag J., Chahal P.S., Kamen A.A. (2019). Achieving High-Yield Production of Functional AAV5 Gene Delivery Vectors via Fedbatch in an Insect Cell-One Baculovirus System. Mol. Ther. Methods Clin. Dev..

[27] Pereira Aguilar P., González-Domínguez I., Schneider T.A.T.A., Gòdia F., Cervera L., Jungbauer A., González-Domínguez I., Schneider T.A.T.A., Gòdia F., Cervera L. (2019). At-line multi-angle light scattering detector for faster process development in enveloped virus-like particle purification. J. Sep. Sci..

[28] Cervera L., Fuenmayor J., González-Domínguez I., Gutiérrez-Granados S., Segura M.M., Gòdia F. (2015). Selection and optimization of transfection enhancer additives for increased virus-like particle production in HEK293 suspension cell cultures. Appl. Microbiol. Biotechnol..

[29] Carvalho S.B., Silva R.J.S., Moreira A.S., Cunha B., Clemente J.J., Alves P.M., Carrondo M.J.T., Xenopoulos A., Peixoto C. (2019). Efficient filtration strategies for the clarification of influenza virus-like particles derived from insect cells. Sep. Purif. Technol..

[30] Malvern Instruments Limited (2015). Determining Fluorescence Limit of Detection with Nanoparticle Tracking Analysis (NTA).

[31] González-Domínguez I., Puente-Massaguer E., Cervera L., Gòdia F. (2020). Quantification of the HIV-1 virus-like particle production process by super-resolution imaging: From VLP budding to nanoparticle analysis. Biotechnol. Bioeng..

[32] Anderson E.C., Petersen D.F., Tobey R.A. (1970). Density Invariance of Cultured Chinese Hamster Cells with Stage of the Mitotic Cycle. Biophys. J..

[33] Westoby M., Chrostowski J., De Vilmorin P., Smelko J.P., Romero J.K., Carolina N. (2011). Effects of Solution Environment on Mammalian Cell Fermentation Broth Properties: Enhanced Impurity Removal and Clarification Performance. Biotechnol. Bioeng..

[34] Nestola P., Villain L., Peixoto C., Martins D.L., Alves P.M., Carrondo M.J.T., Mota J.P.B. (2014). Impact of grafting on the design of new membrane adsorbers for adenovirus purification. J. Biotechnol..

[35] McNally D.J.J., Darling D., Farzaneh F., Levison P.R.R., Slater N.K.H.K.H. (2014). Optimised concentration and purification of retroviruses using membrane chromatography. J. Chromatogr. A.

[36] Kutner R.H., Puthli S., Marino M.P., Reiser J. (2009). Simplified production and concentration of HIV-1-based lentiviral vectors using HYPERFlask vessels and anion exchange membrane chromatography. BMC Biotechnol..

[37] Nestola P., Peixoto C., Silva R.R.J.S., Alves P.M., Mota J.P.B., Carrondo M.J.T. (2015). Improved virus purification processes for vaccines and gene therapy. Biotechnol. Bioeng..

[38] Venereo-Sanchez A., Simoneau M., Lanthier S., Chahal P., Bourget L., Ansorge S., Gilbert R., Henry O., Kamen A. (2017). Process intensification for high yield production of influenza H1N1 Gag virus-like particles using an inducible HEK-293 stable cell line. Vaccine.

[39] Knezevic I., Stacey G., Petricciani J. (2008). WHO Study Group on cell substrates for production of biologicals, Geneva, Switzerland, 11–12 June 2007. Biologicals.

[40] Eaton L.C. (1995). Host cell contaminant protein assay development for recombinant biopharmaceuticals. J. Chromatogr. A.

