# Supplementary material for: A Four-Step Purification Process for Gag VLPs: From Culture Supernatant to High-Purity Lyophilized Particles
Source: Vaccines (Basel). 2021 Oct 9;9(10):1154. doi: 10.3390/vaccines9101154 (PMC8539588; doi:10.3390/vaccines9101154)
Supplement: Supplementary file 1 [file vaccines-09-01154-s001.zip › vaccines-1334000-supplementary.pdf]

Supplementary Materials

A Four-Step Purification Process for Gag VLPs: From Culture Supernatant to High-Purity Lyophilized Particles

Irene González-Domínguez [1,*,†], Elianet Lorenzo [1], Alice Bernier [2], Laura Cervera [1], Francesc Gòdia [1] and Amine Kamen [2]

1 Departament d'Enginyeria Química Biològica i Ambiental, Universitat Autònoma de Barcelona, 08193 Barcelona, Spain; Elianet.Lorenzo@uab.cat (E.L.); Laura.Cervera@uab.cat (L.C.); Francesc.Godia@uab.cat (F.G.)

2 Department of Bioengineering, McGill University, Montreal, QC H3A 0E9, Canada; bernier.alice@videotron.ca (A.B.); amine.kamen@mcgill.ca (A.K.)

* Correspondence: irene.gonzalez.dominguez.phd@gmail.com

† Present address: Department of Microbiology, Icahn School of Medicine at Mount Sinai, New York, NY 10029, USA.

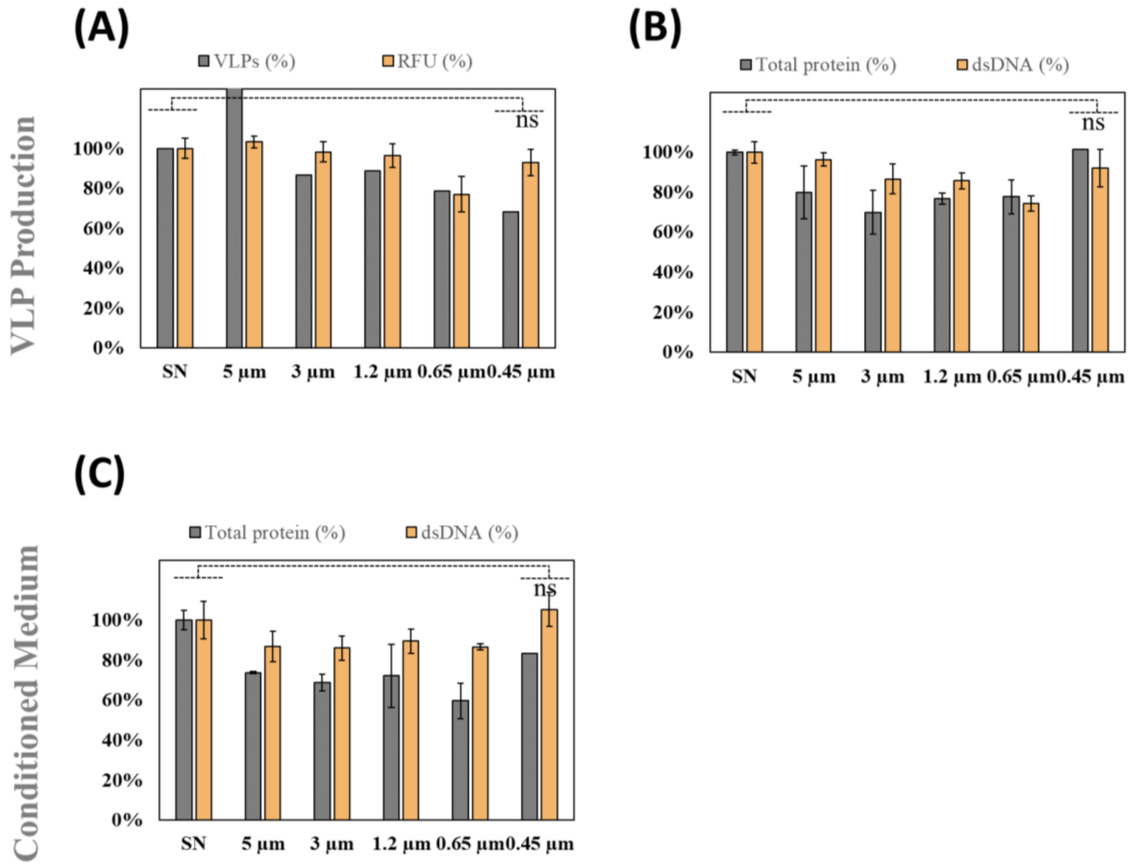

Supplementary Figure S1: Sequential filtration experiments of Gag-eGFP VLPs. (A-B) Analysis of Gag-eGFP VLP productions: (A) VLP nanoparticle concentration measured by flow virometry (VLPs) and relative fluorescent units (RFU) and (B) total protein and dsDNA content after the different filters. (C) Analysis of conditioned cell culture medium in regard to total protein and dsDNA content after the different filters. Statistical analyses of technical replicates were performed with ANOVA or T Student test. Ns: non-significant.

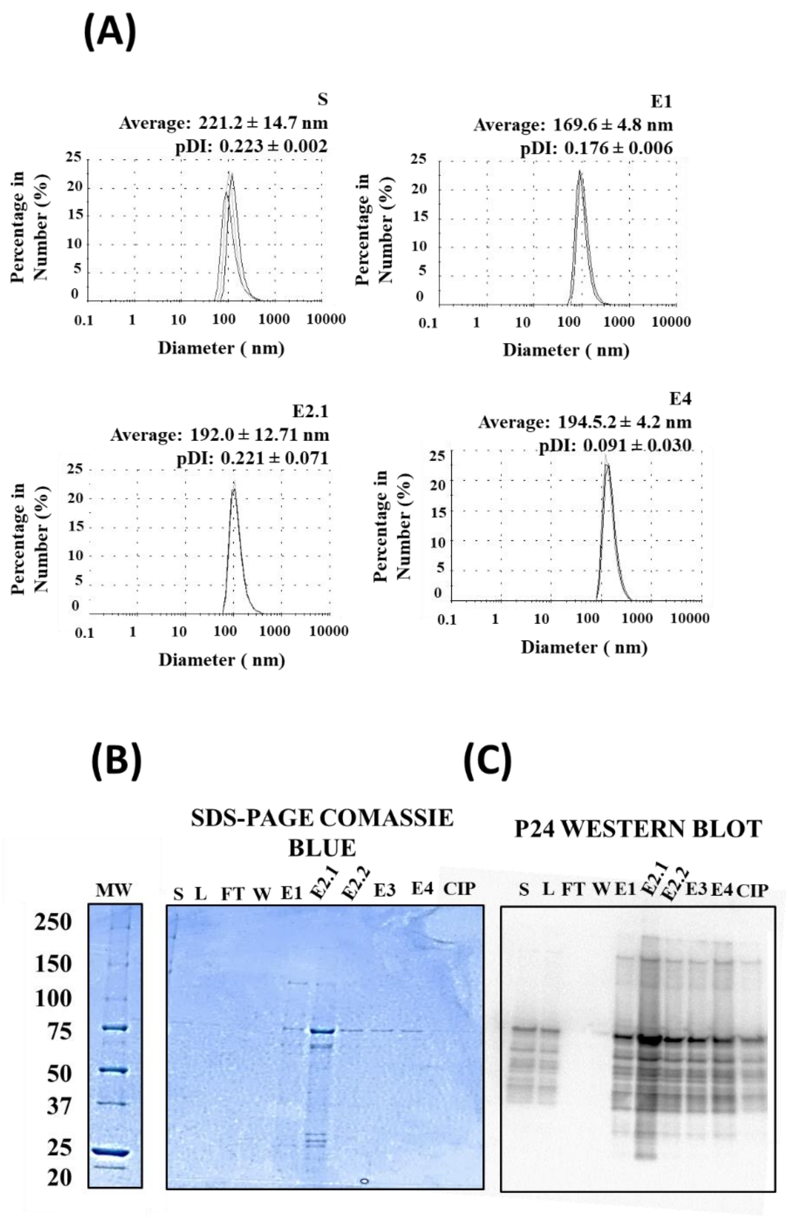

Supplementary Figure S2: Biophysical and biochemical characterization of purified Gag-eGFP VLPs. From chromatogram (Figure 4D), peaks E1, E2.1 and E3 were loaded onto a pd10 desalting column and Gag-eGFP VLPs contained in the void volume were recovered in PBS (HyClone) and further analyzed by DLS, where three independent measurements were performed per sample (A); Average: mean hydrodynamic diameter; pDI: polydispersity index. SDS-PAGE Comassie Blue (B) and p24 Western Blot (C) were also performed from the different fractions obtained in this run. S: supernatant; L: load material; FT: flow-through; W: wash; E1-E4: pooled fractions for peaks 1-4 and CIP: cleaning in place with 2M NaCl.
